# Fibroblast Growth Factor 7 Suppresses Fibrosis and Promotes Epithelialization during Wound Healing in Mouse Fetuses

**DOI:** 10.3390/ijms23137087

**Published:** 2022-06-25

**Authors:** Kento Takaya, Noriko Aramaki-Hattori, Shigeki Sakai, Keisuke Okabe, Toru Asou, Kazuo Kishi

**Affiliations:** Department of Plastic and Reconstructive Surgery, Keio University School of Medicine, Tokyo 160-8582, Japan; nonken@2001.jukuin.keio.ac.jp (N.A.-H.); shigekix724@hotmail.com (S.S.); dawndawn@hotmail.co.jp (K.O.); mori@ideajapan.com (T.A.); kkishi@a7.keio.jp (K.K.)

**Keywords:** fibroblast growth factor, skin regeneration, mouse, fibrosis, scar

## Abstract

Adult mammalian wounds leave visible scars, whereas skin wounds in developing mouse fetuses are scarless until a certain point in development when complete regeneration occurs, including the structure of the dermis and skin appendages. Analysis of the molecular mechanisms at this transition will provide clues for achieving scarless wound healing. The fibroblast growth factor (FGF) family is a key regulator of inflammation and fibrosis during wound healing. We aimed to determine the expression and role of FGF family members in fetal wound healing. ICR mouse fetuses were surgically wounded at embryonic day 13 (E13), E15, and E17. Expression of FGF family members and FGF receptor (FGFR) in tissue samples from these fetuses was evaluated using in situ hybridization and reverse transcription-quantitative polymerase chain reaction. *Fgfr1* was downregulated in E15 and E17 wounds, and its ligand *Fgf7* was upregulated in E13 and downregulated in E15 and E17. Recombinant FGF7 administration in E15 wounds suppressed fibrosis and promoted epithelialization at the wound site. Therefore, the expression level of *Fgf7* may correlate with scar formation in late mouse embryos, and external administration of FGF7 may represent a therapeutic option to suppress fibrosis and reduce scarring.

## 1. Introduction

The skin constitutes the largest organ in the human body and covers the entire surface of the body. Skin injury is repaired by a complex healing process that includes blood coagulation, inflammation, re-epithelialization, granulation tissue formation, and finally tissue remodeling. Under normal conditions, the wound heals but lacks all skin appendages, leaving a visible scar that exhibits decreased tensile strength and elasticity [1,2]. Since surgical procedures cannot completely erase the scar, and the impaired healing leads to considerable morbidity and imposes an enormous economic burden on the health care system, studies have focused on the development of therapies that can completely regenerate the skin. In contrast, fetal skin wounds that are created before a certain developmental stage regenerate without leaving scars [3,4,5]. The regeneration ability of fetuses has been demonstrated in various tissues, and the precise understanding of this phenomenon may facilitate the development of a model for complete tissue regeneration. Elucidating the molecular mechanisms that occur during the transition from complete skin regeneration in early development to a scar-forming phenotype, similar to that of adult animals, in late development is of great interest.

In general, the wound repair process is regulated by several growth factors and cytokines [6]. Factors of particular importance are fibroblast growth factors (FGFs), which comprise a family of 22 polypeptides. Most of them exert their functions via the activation of four receptor-type tyrosine kinases called FGFR1–4 [7,8]. Various members of the FGF family have been reported to contribute to the wound repair process; FGF2 is particularly important for wound angiogenesis and granulation tissue formation, and the ligands that activate FGF receptors on keratinocytes regulate re-epithelialization [9]. FGFR2-IIIb, expressed in keratinocytes, is activated by FGF1, FGF7, FGF10, and FGF22, which are expressed in normal skin wounds [10]. In addition, FGF1, FGF10, and FGF22 activate FGFR1-IIIb, another receptor expressed in keratinocytes [11]. Furthermore, FGFR-1 and FGFR-2 are known to represent important regulators of keratinocyte migration in vitro and in wounded skin [12,13]. However, owing to its cumbersome classification, the contribution of specific FGF receptors and their ligands to wound re-epithelialization has not yet been determined and the underlying mechanisms have not been characterized.

In our originally developed mouse embryonic wound healing model, we found that wounds up to embryonic day 16 (E16) fully regained their dermal structure, but in embryos and adults after E17, the wounded area lost its original skin structure, which was replaced by fibrotic scar tissue composed of collagen fibers [14]. We also found that wounds up to E13 completely regenerated their structure and appearance, including epidermal texture, and healed without a scar, while fetal wounds inflicted after E14 left visible scars. We hypothesized that scar formation can be controlled by regulating the expression of FGF family members in embryonic mouse wounds during development. Therefore, we aimed to investigate the expression and role of the FGF gene family in fetal wound healing. We focused on three time points during development, namely E13, where all structures are completely regenerated, E15, where visible scars remain but dermal structures are regenerated, and E17, where tissue is replaced by scars as in adult animals, and compared the differences. We found that FGF7 may correlate with scar formation in late mouse embryos, and external administration of FGF7 may assist in the development of therapies for complete skin regeneration.

## 2. Results

### 2.1. Expression of FGFR Members during Fetal Wound Healing in Mice

**Figure 1:**
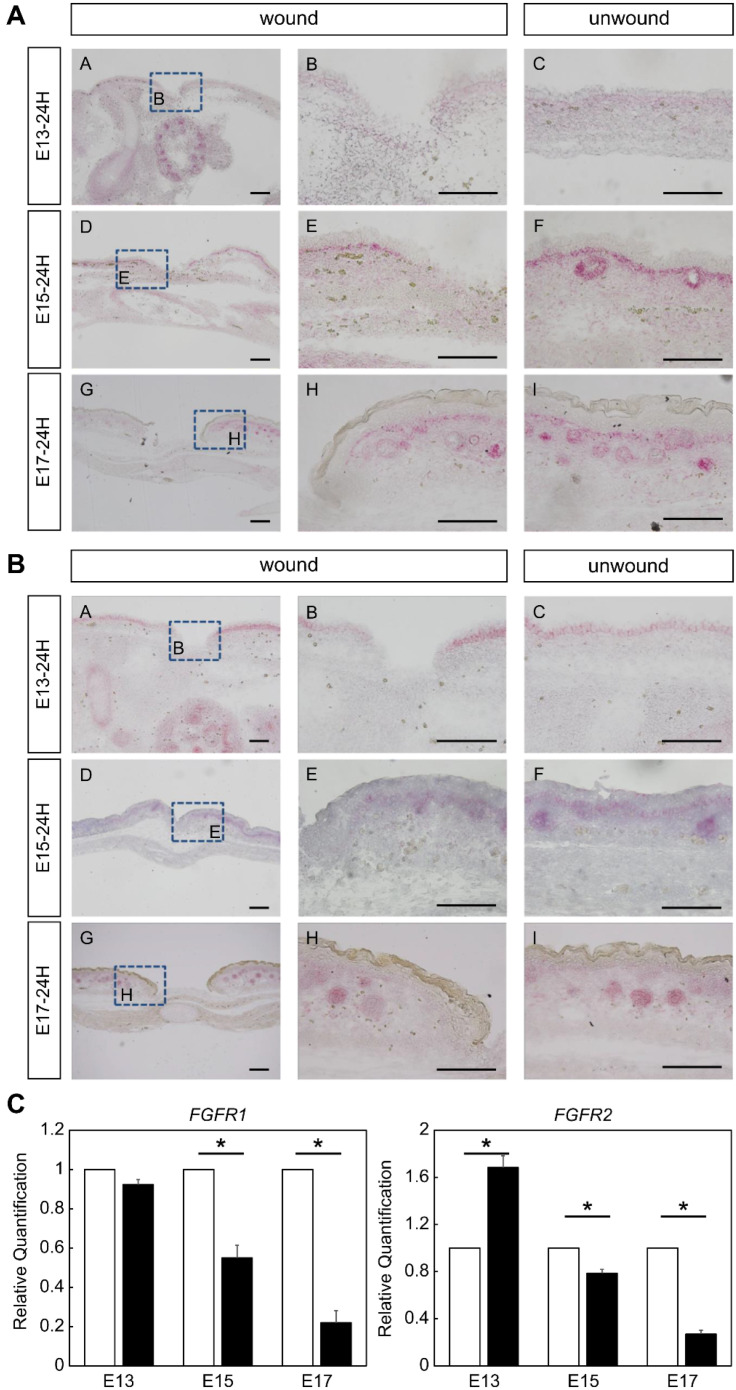
Expression of FGFRs during the wound healing process in mouse fetuses. FGFR1,2 positivity is indicated by red color. (**A**) in situ hybridization of FGFR1. (**B**) in situ hybridization of FGFR2. Bar = 100 µm. (**C**) Real-time polymerase chain reaction analysis of gene expression of *Fgfr1* and *Fgfr2*. *, *p* < 0.05. FGFR, fibroblast growth factor receptor; E13, embryonic day 13.

First, we confirmed the expression of FGFR1 and FGFR2, which are receptors of the FGF family, during fetal wound healing in mice (Figure 1). At E15 and E17, FGFR1 was specifically expressed in the basal layer and hair follicles and its expression tended to decrease around the wound area (Figure 1A).

FGFR2 was also specifically expressed in the basal layer in normal skin at E13, and in the basal layer and hair follicles at E15 and E17. In both periods, FGFR2 expression was attenuated around the wound (Figure 1B). Real-time polymerase chain reaction (PCR) based on RNA extracted via laser microdissection (LMD) of the wound showed that gene expression of *Fgfr1* alone was significantly decreased in the wound at E15 and E17 (E15, *p* = 0.019; E17, *p* = 0.006). For *Fgfr2*, the gene expression was significantly decreased in E13 wounds and was upregulated compared to that in the control (*p* = 0.02), and gene expression was decreased in E15 and E17 wounds (E15, *p* = 0.022; E17, *p* = 0.00019) (Figure 1C). Thus, the expression pattern of *Fgfr* was found to change between the periods when wounds regenerate and when they no longer regenerate.

### 2.2. Expression of FGF Family Members during Fetal Wound Healing in Mice

**Figure 2:**
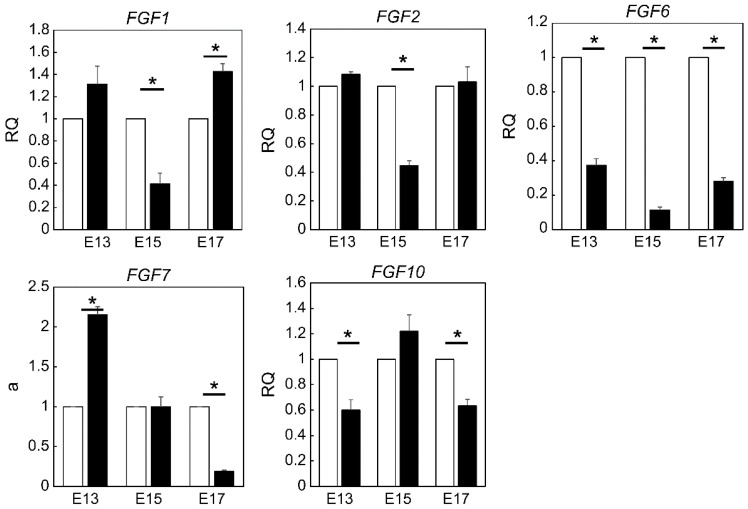
Gene expression of FGF family members during the wound healing process in mouse fetuses. *, *p* < 0.05. RQ, relative quantification. FGF, fibroblast growth factor 7.

Gene expression of *Fgf1, Fgf2, Fgf3, Fgf4, Fgf5, Fgf6, Fgf7, Fgf8, Fgf9, Fgf10*, and *Fgf17*, and ligands of FGFR was measured via real-time PCR (Figure 2). The expression of *Fgf3, Fgf4, Fgf5, Fgf8, Fgf9*, and *Fgf17* was detected in trace amounts; *Fgf1* expression was significantly increased in the wounds at E17 (*p* = 0.025) and significantly decreased at E15 (*p* = 0.026) compared to that in normal skin. *Fgf2* expression was decreased in the wound at E15 (*p* = 0.0039). *Fgf6* expression was decreased in E13 (*p* = 0.0001), E15 (*p* = 0.0003), and E17 (*p* = 0.0008) wounds compared to that in normal skin. *Fgf7* expression was significantly increased in E13 wounds (*p* = 0.0077) with no difference in expression in E15 wounds and significantly decreased in E17 wounds (*p* = 0.00047) compared to that in normal skin. *Fgf10* expression was decreased in the wound at E13 (*p* = 0.039) and E17 (*p* = 0.018); however, there was no difference in expression at E15 compared to that in normal skin.

### 2.3. Role of FGF7 in the Wound Healing Process in Mouse Fetuses

**Figure 3:**
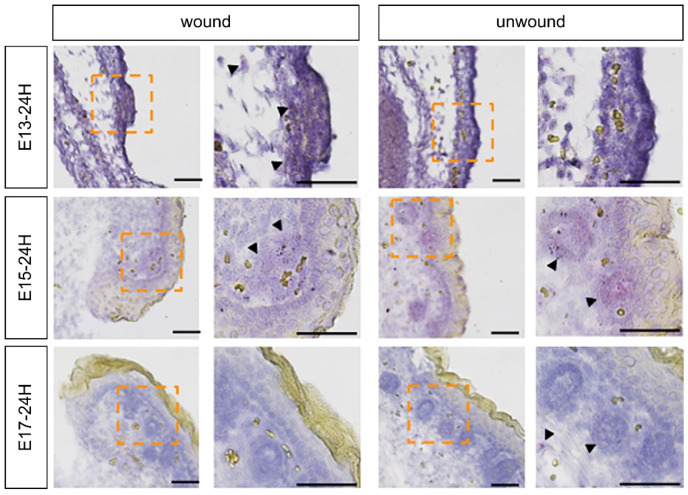
FGF7 expression during the wound healing process in mouse fetuses determined via in situ hybridization. FGF, fibroblast growth factor 7; E13, embryonic day 13.

Since *Fgf7* was upregulated in E13 and downregulated thereafter, coinciding with periods of complete skin regeneration and non-regeneration, we hypothesized that this may influence wound healing. In situ hybridization analysis showed that FGF7 expression was enhanced in E13 wounds compared to that in the normal area. In E15 wounds, no change in expression was observed between the wounded and normal areas; however, the expression itself was found to be attenuated in E17 wounds compared to that in E13 and E15 wounds (Figure 3).

**Figure 4:**
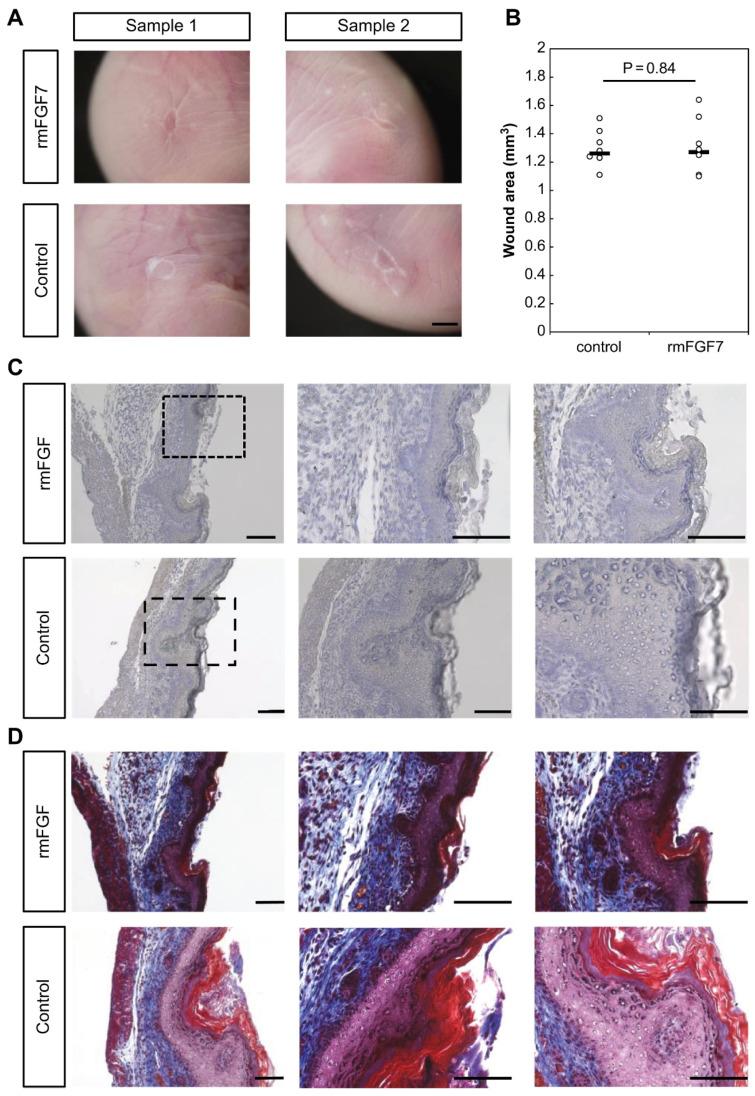

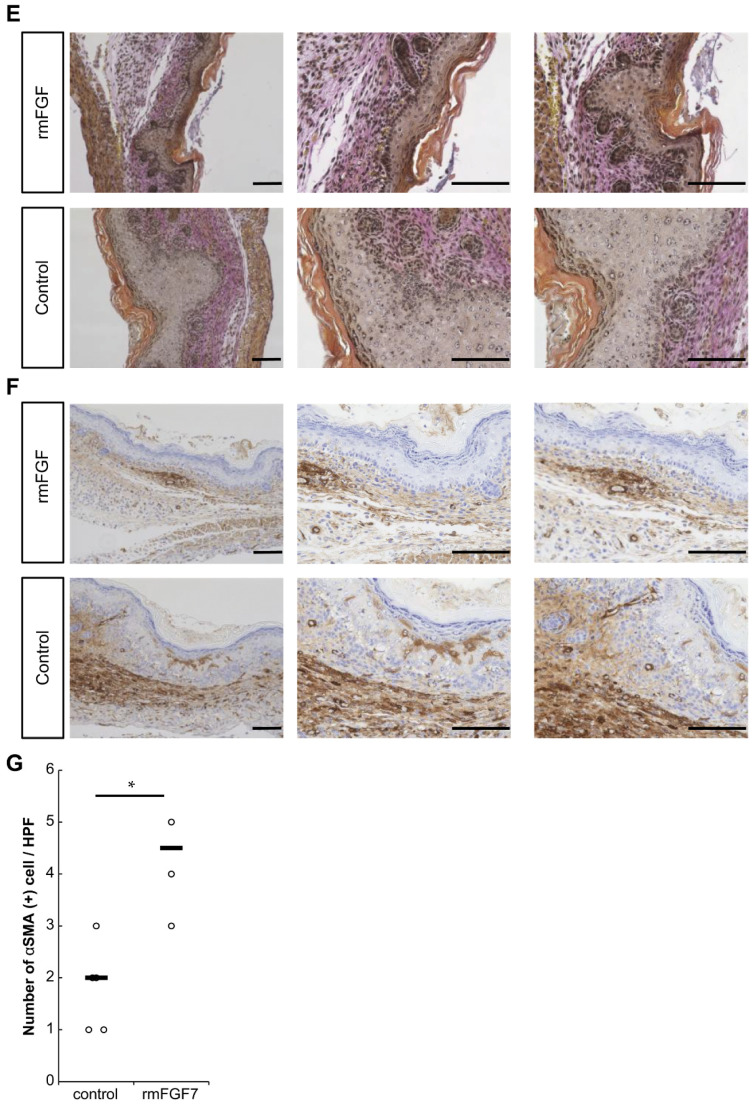
Effect of rmFGF7 administration on wound healing in mouse fetuses. (**A**) Macrograph of a scar; bar = 1 mm. (**B**) Comparison of the wound area. (**C**) Hematoxylin-eosin staining of wounds; bar = 100 µm. (**D**) Masson trichrome staining of the wound. Bar = 100 µm. (**E**) Elastica van Gieson staining of the wound; bar = 100 µm. (**F**) Immunostaining of the wound for αSMA; bar = 100 µm. (**G**) Counting of αSMA-positive cells. *, *p* < 0.05. HPF, high power field; FGF7, fibroblast growth factor 7; αSMA, alpha-smooth muscle actin.

To investigate the effect of FGF7 on wound healing, recombinant FGF7 (rmFGF7) was administered to E15 fetuses at the time of wounding for observation. No effect on scar morphology and no significant difference in wound area were found (Figure 4A,B). Histological observation of the sections showed that the epidermis was curved and formed scars in the control group, whereas the epidermis formation was accelerated in the rmFGF7-treated group (Figure 4C). Masson trichrome staining showed that collagen fibers were densely formed in the wound area in the mFGF7-treated group (Figure 4D). Elastica van Gieson’s staining showed that the expression of myofibers was enhanced at the wound margin in the rmFGF7-treated group. In the control group, staining was enhanced throughout the wound and was found to be extensively enhanced in the dermis (Figure 4E). Alpha smooth muscle actin (αSMA) staining showed significantly fewer α-SMA-positive cells in the rmFGF7 group (*p* = 0.00000066) than in the control group (Figure 4F,G). Therefore, fibrosis occurred only at the wound margins in the rmFGF7 group, whereas fibrosis occurred over a wide area of the dermis in the wound in the control group. In summary, administration of rmFGF7 suppressed fibrosis in the wound and promoted epidermis formation.

## 3. Discussion

Despite the fact that prominent scarring greatly affects the quality of life of patients [15], there is no method available to regenerate and heal scars without a trace after injury. Therefore, there is an urgent need to develop novel and efficient strategies for the treatment of wounds, which requires a thorough understanding of the underlying cellular and molecular mechanisms.

Using a proprietary fetal mouse wound healing model, we focused on one of the factors involved in wound healing, the FGF family, and compared their expression in wounds during the transition from regeneration to repair. Genes encoding FGFR1 and FGFR2, which are receptors of the FGF family, were downregulated in the wound after E15. The expression of *Fgf7*, which encodes a ligand that binds to FGFR1 and FGFR2, increased at E13 and decreased after E15. Intra-amniotic administration of rmFGF7 inhibited scar formation, as observed histologically, suggesting that FGF7 may play a role in skin regeneration. Normally, 24 h after injury corresponds to the inflammatory phase of wound healing, but wound healing in fetuses is faster than that in adult animals and is completed in about 48 h [14]. Therefore, the 24-h time point observed in this study corresponds to the proliferative or reconstructive phase of wound healing, suggesting that the behavior of the FGF family may have influenced scar formation. It is known that the degree of scarring correlates with the number of αSMA-positive cells [16], and the administration of rmFGF7 significantly reduced the number of αSMA-positive cells in the scar area. In summary, our results indicate that administration of rmFGF7 may be able to control scar formation to a certain extent.

With regard to FGFR expression, a previous study showed that loss of FGFR1 and FGFR2 in keratinocytes results in a defect in wound healing similar to the abnormality seen in chronic human skin ulcers, and this phenotype is characterized by impaired severe re-epithelialization combined with decreased wound contraction [17]. Loss of FGFR1 and FGFR2 in keratinocytes strongly affects dermal immune cells and fibroblasts due to strong upregulation of S100A8, S100A9, and interleukin-1 family member 8 [13]. However, these factors do not affect the differentiation of myofibroblasts, which play a particularly important role in wound contraction [18]; rather, changes in the connective tissue that occur in these mice are likely responsible for the wound contraction defect [19].

Regarding the FGF family of ligands, the well-studied FGF2 inhibits transforming growth factor-beta 2 (TGFβ)-mediated fibroblast activation, and treatment with exogenous FGF2 also inhibits α-SMA, calponin, transgelin, connective tissue growth factor, and ED-A fibronectin expression. FGF2 and FGF7 are known to inhibit myofibroblast migration and activation by attenuating TGFβ-mediated increases in collagen I expression [20]. FGF2 and 7 have been reported to behave similarly as fibroblast growth factors [21], and this study suggests that FGF7, like FGF 2, is a novel target for the inhibition of fibrosis.

A limitation of this study is that the downstream signaling and the target cells of FGF7 were not investigated. Scar healing is generally attributed to the presence of inflammatory cells or myofascial-derived fibroblasts at the base of E17 embryonic wounds, which are reported to result in scar formation. We previously found that dermal mesenchymal cells are responsible for the remodeling of dermal structures in the wound and that after E17, scar formation is mediated by mesenchymal cells of fascial origin [22]. Thereafter, the classification and behavior of skin mesenchymal cells, that is undifferentiated fibroblasts, have been reported to play an important role in wound healing [23,24], and the behavior of FGF7 and these cells requires further investigation. Future studies should determine whether this phenomenon occurs in mammals other than mice. Moreover, additional experiments are required in the future to determine whether wound healing changes in an FGF7 concentration-dependent manner and to determine the optimal concentration. Furthermore, the timing of FGF7 administration and signaling should be investigated to determine changes in FGFR expression over time after administration.

Based on the present data showing a correlation between FGF7 expression and non-scarring skin regeneration during development, the application of this phenomenon in human skin and wound tissues may be useful for the development of scarless wound healing and regenerative medicine.

## 4. Materials and Methods

### 4.1. Ethical Consideration

The research protocol was reviewed and approved by the Institutional Animal Care and Use Committee of Keio University School of Medicine (approval number: 20170914). All experiments were conducted in accordance with the institutional guidelines for animal experiments at Keio University. These experiments have been reported in accordance with the Reporting of In Vivo Experiments on Animals guidelines.

### 4.2. Fetal-Wounding Procedure

Eight-week-old female Institute of Cancer Research (ICR) mice were used in this experiment. All mice were obtained from Sankyo Laboratory Services (Shizuoka, Japan). Vaginal plugs were checked twice daily. When a plug was observed, the fetus was designated E0; the fetus was wounded at E13, E15, and E17. Surgeries were performed on five pregnant mice per time point. Pregnant mice were anesthetized using 3% isoflurane, and the abdominal wall was incised to expose the uterus. Using an operating microscope, the myometrium and the amniotic and yolk sacs were incised. Subsequently, using surgical micro-scissors, a full-layer incision of approximately 2 mm in length was made in the lateral thoracic region of the fetus. In E13 after wounding, the amnion and yolk sac were sutured with 9–0 nylon, while the myometrium was left open and unsutured to prevent possible uterine rupture and fetal death resulting from the high internal pressure during development. The fetus was returned to the abdominal cavity with the amnion and yolk sac covered and the myometrium uncovered, and the abdomen was closed; in E15 and E17, after making the fetal wound, the myometrium was sutured with 9–0 nylon, the uterus was returned to the abdominal cavity, and the abdomen was closed. Subsequently, just before the closure of both wounds, 1 μg/g bodyweight of the uterine relaxant ritodrine hydrochloride (Fujifilm Wako Pure Chemical, Osaka, Japan) was administered intraperitoneally. The peritoneum and skin were then sutured with 5–0 nylon thread. Maternal mice were euthanized via cervical dislocation, and fetuses were harvested 24 h after wounding. Wounds were made in at least four fetuses. Fetal skin was harvested and fixed in 4% paraformaldehyde for 24 h and the fixed tissue was embedded in paraffin and stained.

### 4.3. Immunohistochemistry

Paraffin-embedded specimens were sliced into 7 µm-thick sections and mounted on glass slides. After drying overnight at room temperature (20 °C) to allow the specimens to adhere to the slides, paraffin was dissolved using a slide heater (ThermoBrite; Leica Biosystems, Nussloch, Germany) at 65 °C for 30 min immediately before use. The slides were then deparaffinized by changing xylene twice at room temperature (5 min each). Slides were transferred twice to 100% ethanol (3 min each), once to 95%, 70%, and 50% ethanol (3 min each), and rehydrated at room temperature. After antigen activation in response to heat, the samples were incubated with 2% goat serum in phosphate-buffered saline (PBS) for 30 min at room temperature to block nonspecific binding sites. The cells were then incubated overnight at 4 °C with the antibody anti-αSMA (ab5693; Abcam, Cambridge, United Kingdom) diluted 1:100 in PBS. After washing three times with PBS, the cells were incubated with a 1:500 dilution of an anti-mouse horseradish peroxidase-labeled rabbit IgG reagent ImmPRESS (Vector Laboratories, Burlingame, CA, USA) in PBS for 1 h at room temperature. Signals were amplified using the avidin-biotinylated peroxidase complex (ABC) method using the VECTASTAIN ABC kit (Vector Laboratories) and incubated in 20 mg/dL 3,3′-diaminobenzidine solution (Fujifilm Wako Pure Chemical) for 1–3 min. The sections were then washed once for 5 min with running tap water before counterstaining the nuclei for 6 s at room temperature with Gill’s hematoxylin solution (Merck Millipore, Billerica, MA, USA). Finally, the sections were rinsed with tap water for 5 min, dehydrated four times with ethanol (95%, 95%, 100%, and 100% for 5 min each), rinsed with xylene three times, and sealed with Mount Quick Sealant (Takara Bio, Shiga, Japan). Slides were observed using an integrated stereomicroscope (BZ-X800; Keyence, Osaka, Japan). The number of positive cells in the high power field at 400× magnification under the microscope was counted for 10 fields per slide. The experiment was repeated three times.

### 4.4. In Situ Hybridization

In situ hybridization analysis was performed using the QuantiGene ViewRNA ISH Tissue Assay (Thermo Fisher Scientific, Waltham, MA, USA) according to the manufacturer’s protocol and as previously reported [25]. Briefly, paraffin sections were dried at 60 °C for 60 min and paraffin was removed using Histo Clear (National Diagnostics, Atlanta, GA, USA) and 100% ethanol. A hydrophobic wall was created around the tissues using ImmEdge Pen (Vector Laboratories). As pretreatment, the tissue was boiled in pretreatment solution for 5 min, rinsed, and treated with protease solution at 40 °C for 20 min. After washing twice with PBS, the samples were fixed in 10% neutral-buffered formalin solution for 5 min and washed again with PBS. The target probe was diluted 50-fold in a probe set diluent QF solution warmed to 40 °C and incubated at 40 °C for 3 h. After washing three times with wash buffer, the preamplifier solution was incubated at 40 °C for 25 min. The preamplifier solution was washed three times with wash buffer again and incubated at 40 °C for 15 min. The AP enhancer solution was decanted and Fast Red Tablet was dissolved in napthol buffer and incubated at room temperature for 5 min. After decanting the AP enhancer solution, Fast Red Tablet was dissolved in Napthol buffer and incubated at 40 °C for 30 min. After washing the samples twice with PBS, nuclear staining was performed with Gill’s Hematoxylin solution and washed with water three times. After removing excess staining solution with 0.01% ammonia solution, the samples were rinsed with water again and sealed in Ultramount Aqueous Permanent Mounting Medium (Agilent Technologies, Santa Clara, CA, USA). FGFR1 (VB6-3197928-VT), FGFR2 (VB6-18570-VT), and FGF7 (VB1-3029756-VT) were used as probes.

### 4.5. LMD, RNA Isolation, and Reverse Transcription

LMD was performed using a PALM MicroBeam (Carl Zeiss, Oberkochen, Germany). The manufacturer’s recommended slides and collection tubes (AdhesiveCap 500 opaque, Carl Zeiss) were set up, and the tissue was carefully cut after adjusting the aperture and intensity using a 20× magnification objective lens. The tube caps were filled with Buffer RLT (RNeasy Micro Kit; Qiagen, Hilden, Germany) and β-mercaptoethanol to allow the separation of intact RNA. Total RNA was extracted from cells or skin tissues using a monophasic solution of phenol and guanidine isothiocyanate (ISOGEN; NipponGene, Tokyo, Japan) according to the manufacturer’s instructions. Total RNA was mixed with a random primer, reverse transcriptase, and dNTP mixture (Takara Bio, Tokyo, Japan). The mixture was incubated in a T100TM thermal cycler (Bio-Rad Laboratories, Hercules, CA, USA) at 25 °C for 5 min, 55 °C for 10 min, and 80 °C for 10 min to heat inactivate the reverse transcriptase and synthesize cDNA.

### 4.6. Quantitative Real-Time PCR

Quantitative real-time PCR was performed using an Applied Biosystems 7500 Fast Real-Time PCR System (Thermo Fisher Scientific). A total of 40 cycles were performed, and the fluorescence of each sample was measured at the end of each cycle. The PCR reaction was performed in two major steps: holding the reagent at 95 °C for 10 s (denaturation) and at 60 °C for 30 s (annealing and extension). In the subsequent melting curve analysis phase, the temperature was increased from 60 °C to 95 °C and fluorescence was measured continuously. Primers for *Fgf1* (Mm00438906_m1), *Fgf2* (Mm01285715_m1), *Fgf3* (Mm00433290_m1), *Fgf4* (Mm00438917_m1), *Fgf5* (4331182 Mm03053745_s1), *Fgf6* (Mm01183111_m1), *Fgf7* (Mm00433291_m1), *Fgf8* (Mm00438922_m1), *Fgf9* (Mm00438922_m1), *Fgf10* (Mm00433275_m1), *Fgf17* (Mm00433280_m1), *Fgfr1* (4331182 Hs00915142_m1), and *Fgfr2* (Hs01552926_m1) were used (all Thermo Fisher Scientific). PCR master mix (Cat. 4352042; Applied Biosystems, Foster City, CA, USA) was used according to the manufacturer’s instructions. *Actb* (Mm02619580_g1) was used as a control gene for normalization according to the manufacturer’s instructions. Gene expression levels at normal sites were used as the baseline, and the fold-change values were determined using the 2^−ΔΔCt^ method.

### 4.7. rmFGF Administration into Mouse Fetuses

ICR mice on day 15 of gestation were operated on in the same manner as described above to generate wounds. Simultaneously, 50 µL of rmFGF7 (R&D Systems, Minneapolis, MN, USA) diluted in PBS to 5 µg/mL was administered per fetus. Doses were determined based on previous reports [26]. An equal volume of PBS was administered as a control. Wounds were made in 8 fetuses in each group. After 48 h, the fetuses were collected. The wounds in the fetuses were harvested, macroscopically observed, and fixed with 4% paraformaldehyde. The skin of the wound was excised and embedded in paraffin to prepare 7 µm-thick paraffin sections. The sections were stained with hematoxylin and eosin [27], Masson trichrome [28], and Elastica Wang–Gieson stains [29].

### 4.8. Statistical Analysis

Mann–Whitney U tests were performed to determine the significance of differences in gene expression using Statistica software version 9.0 (StatSoft, Tulsa, OK, USA). Results of descriptive statistics are presented as the mean ± standard deviation. The threshold for significance was set at *p* < 0.05. Each experiment was performed in triplicate.

## Data Availability

The data presented in this study are available on request from the corresponding author.
